# Factors associated with COVID-19 in children aged 0 to 15 in Niger, 2020

**DOI:** 10.11604/pamj.2024.47.117.41490

**Published:** 2024-03-12

**Authors:** Habibatou Idé Amadou, Herman Yoda, Dieudonné Tialla, Pauline Kiswendsida Yanogo, Djibril Barry, Maman Laminou Ibrahim, Samaila Aboubacar, Abdoul Salam Youssoufou Souley, Abdoulaye Ousmane, Nicolas Meda

**Affiliations:** 1 Burkina Field Epidemiology and Laboratory Training Program, Joseph KI-ZERBO University, BP 9268, Ouagadougou, Burkina Faso,; 2 Institute of Research in Health Sciences, BP 7047 Ouagadougou 3, Burkina Faso,; 3 Faculty of Medicine, University of Ouagadougou 1 Joseph KI-ZERBO, BP 7021 Ouagadougou, Burkina Faso,; 4 Medical and Health Research Center, BP 10887, Niamey, Niger,; 5 Faculty of Health Sciences, Abdou Moumouni University of Niamey, BP 10146 Niamey, Niger,; 6 Amirou Boubacar Diallo National Hospital, BP 10146, Niamey, Niger,; 7 Faculty of Health Sciences, University Dan Dicko Dankoulodo of Maradi, Maradi, Niger

**Keywords:** Factors, COVID-19, children, database, Niger

## Abstract

On January 30, 2020, the WHO declared COVID-19 a global health emergency. Children were affected in less severe forms. Niger had implemented measures in a context where children were a source of contamination. The aim was to determine the factors associated with COVID-19 in children in Niger from February to August 2020 through an analysis of the national database. We conducted an analytical cross-sectional study including all COVID-19 suspects in the database. We used Excel and Epi Info 7.2.4. software for data extraction and analysis. Frequencies and proportions were calculated, and in a logistic regression, we estimated the ORs of association with their 95% confidence intervals, the factors associated with COVID-19 at the threshold of p<0.05. Of 572 notified cases of suspected COVID-19 in children aged 0-15, 11.36% were positive. The median age of infected children was 10 years [IQR: 5- 13 years]. The male/female sex ratio was 2.1. Children aged 11 to 15 accounted for 49.2%, 61.5% lived in Niamey, 4.6% had comorbidities. The notion of travel was 12.3% and 40% had a notion of contact, 24.4% had a fever, 23.2% had a cough, 18% were hospitalized, and a case-fatality rate of 1.5%. In etiological analysis, the factors associated with COVID-19 were sex ORa=0.51 [0.28-0.93] p=0.028, presence of symptoms ORa=2.29 [1.23-4.25] p=0.008 and notion of contact ORa=0.32 [0.13-0.77] p=0.011. Exposed children were sensitive to COVID-19, and all age groups were affected, with a predominance of males. We recommend barrier measures adapted to young people, and early detection and management of infected children.

## Introduction

Since December 2019, an epidemic of coronavirus 2019 (COVID-19) has emerged in China. The causative virus has been designated as severe acute respiratory syndrome coronavirus 2 (SARS-CoV-2) and is transmitted mainly by respiratory droplets and close contact [1]. On January 30, 2020, the WHO declared the disease a global health emergency [2].

Studies carried out in Wuhan had shown that the majority of COVID-19 patients were adult males, with mean ages of 55.5, 49 and 56 years [3]. Nevertheless, pediatric studies of COVID-19 have been published in recent months, revealing less severe clinical signs in children than in adults [4]. Most pediatric cases were asymptomatic or presented with mild to moderate symptoms or signs. The youngest children (< 1 year) were the most affected by severe forms of the disease, and were the most frequently hospitalized [5]. Age of onset ranged from 1.5 months to 17 years, most of whom had contact with infected cases, often within the family group. Male predominance has been reported in several studies [6]. The prevalence of COVID-19 was 2.4% among under-18s in China, 2.4% among under-15s in Quebec, 1.5% among under-19s in France and 1.7% among under-18s in the USA [5].

After the first confirmed cases in children, special attention was paid to this vulnerable group. [1]. Indeed, young children cannot wear masks and do not benefit from special prevention and control measures. At the same time, children with other illnesses (comorbidities) such as congenital heart, lung and respiratory diseases, malnutrition and tumors are vulnerable to infection by SARS-CoV-2 [2]. COVID-19 is an acute disease with a mortality rate of 2% reported in critically ill children [7]. Africa has so far recorded fewer cases of COVID-19 than China, the USA and Europe [8]. It was reached later than other continents, but by May 3, 2020, all African countries had notified at least one case [9]. As of September 29, 2020, Africa had nearly 1.5 million confirmed cases, with over 35,000 deaths, reported in 55 countries. This represented around 4% of all cases reported worldwide [9]. Available data on COVID-19 in African children are therefore scarce. The majority of studies of infected children come from Asia, Europe and North America. As the epidemiological and clinical contexts in Africa are specific, it is difficult to apply results from these continents to Africa [10].

In Niger, where this coronavirus (SARS-CoV-2)-related epidemic has been raging since March 2020, prevalence among under-16s among confirmed cases was 4.7% with a case-fatality rate of 0.01% as of August 9, 2020 [11]. Like other African countries, Niger was not sufficiently prepared to contain an epidemic of COVID-19. Candidate vaccines were in short supply and reserved for adults. Social distancing measures are difficult to apply because of the communal way of life in Africa, which facilitates closer contact. Although COVID-19 in the pediatric population is rarely severe [7]. However, children are a source of contamination that can spread the disease, especially to the most vulnerable groups, i.e. the elderly and those with comorbidities. It is in this context, and because there is a lack of studies on COVID-19 in children in Niger, that we became interested in determining the factors associated with the disease in 0-15 year-old, with a view to contributing to better control of COVID-19 in children.

## Methods

**Study framework:** Niger is a country in West Africa, situated between Algeria to the north-northwest, Libya to the northeast, Chad to the east, Nigeria to the south, Benin to the south-southwest, Burkina Faso and Mali to the west-southwest. The capital is Niamey. The population was estimated at 22,314,743 in 2019, with an average density of 17.62 inhabitants/km^2^. Children under 15 accounted for 51.32% with an estimated fertility rate of 7.6 children per woman and an estimated life expectancy at birth of 59 years for men and 60 years for women [12].

**Description of study and population:** this was an analytical cross-sectional study with retrospective collection of epidemiological surveillance data for COVID-19 in Niger. The study period spanned six (6) months from February 25 to August 28, 2020. Children aged 0 to 15 suspected of COVID-19 whose data were in the DSRE database were included. We used the data extraction form, which served as a secondary database containing only our variables of interest.

### Field of study

The *Direction de la Surveillance et de la Riposte aux Epidémies (DSRE)* was created by Decree n°2011-21/PRN/MSP of October 26, 2011 and is based in Niamey. It is located at the central level of the health pyramid. Its function is to coordinate epidemiological surveillance activities in the health sector; to prepare the response to epidemics, to disseminate and conserve data relating to epidemiological surveillance of diseases and maternal deaths in the country, to provide ongoing training for health personnel [13].

### Variables

***The dependent variable:*** is the patient's COVID-19 status: “patient COVID-19” coded as “Yes or No”.

***Independent variables:*** children's socio-demographic characteristics: age, gender, origin; exhibition and travel information; the clinic: signs and symptoms; Treatment and progress.

**Data analysis:** the first stage involved the descriptive part, where the frequencies, proportions, averages and extreme values of the variables were calculated. Some of these variables were presented in the form of tables and graphs. In the second part, a univariate analysis was performed. This involved combining the dependent variable, COVID-19 status, with the independent variables. The Odds ratio (OR) with its confidence interval (IC95%) was used to measure the strength of the association. Fisher's corrected Chi 2 was used to test the association between the dependent variable and the independent variables when at least 25% of the cells had a value of less than 5. We also used logistic regression to identify associated factors from multivariate analysis. Variables associated with the occurrence of COVID-19 with a p<20%, were included in a multivariate logistic regression to search for factors independently associated with COVID-19 in children. Variables retained as associated factors were those with a p<0.05. Model fit was determined using the Hosmer-Lemeshow test. The statistical tools used for the analysis were Epi info 7.2.4 and Excel.

**Ethical considerations:** authorization to proceed with data analysis was obtained from the *Direction de la surveillance épidémiologique et la riposte aux épidémies* (0000132/P/AS/DGPS/DSRE of October 26, 2021). Confidentiality was ensured and maintained.

## Results

### Sample description

**Socio-demographic characteristics of COVID-19 suspect children:** a total of 572 suspected COVID-19 cases were enrolled in this study. The median age was 9 years [IQR: 5- 13 years], with extremes ranging from 0 to 15 years. Of the suspected cases, 55.5% were male, with a sex ratio M/F=1.24. The 11-15 age group was the most represented, at 42.8%. Most of the children (62.8%) lived in the Niamey region, followed by Agadez (27.8%).

**Exposure and travel information on COVID-19 suspect children:** exposure factors were history/comorbidities (1.2%), travel by the suspected case (9.4%) and country visited 14 days before admission, of which West Africa was the most visited (48.1%), visit to a healthcare facility 14 days before admission (14.5%), close contact with a person with an acute respiratory infection (8.7%) and contact with a suspected or probable case of COVID-19 (17.7%).

**Characteristics of suspect children by signs and symptoms:** in 24% of cases, the children's symptoms were mainly cough (26.9%), fever (20.5%) and sore throat (11.3%).

**Characteristics of suspected children by mode of care and outcome:** children were followed at home in 9.2% of cases. Suspected children were admitted to hospital in 2% of cases. More than 80% of cases were not reported under this heading, and generally concerned negative cases. The course of the disease was marked by the death of a 9-month-old infant who presented with fever, cough and dyspnea in a severely malnourished situation.

**Figure 1:**
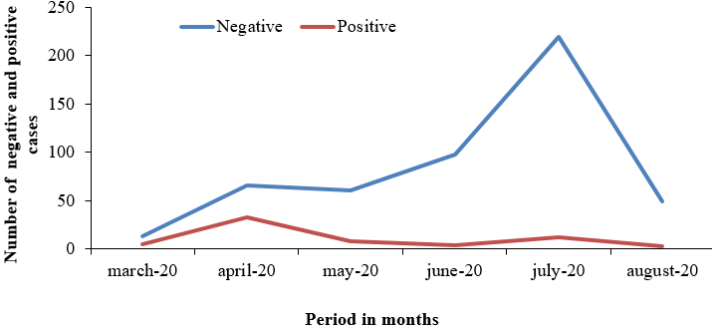
monthly trend of COVID-19 cases among children aged 0-15 - Niger, February - August 2020

**Description of SARS-CoV-2-infected children among suspects:** of the 572 samples tested, only 11% were PCR-positive (n=65). The median age of infected children was 10 years [IQR: 5- 13 years]. There were more positive male cases (67.7%) than female cases (32.5%), i.e. a sex ratio M/F = 2.1. The mean age for girls was 10.71 ± 4.027 years, and for boys 8.07 ± 5.402 years. The monthly trend in COVID-19 among suspected cases showed a peak in COVID-19 in children in April, after the first cases appeared in March. In May, there was a decrease in the number of cases, which remained stable until August (Figure 1).

### Bivariate analyses

**Socio-demographic characteristics of children suspected of having COVID-19 according to PCR test:** COVID-19-positive children were predominantly found in the 11 to 15 age group (49.2%). In univariate analysis, there was no statistically significant association with COVID-19 according to age or residence. However, there was a statistically significant association with COVID-19 according to gender OR=0.55 [0.32 0.96], p=0.036 (Table 1).

**Table 1:** sociodemographic characteristics of suspected cases according to the COVID-19 PCR test in children aged 0-15 years - Niger, February - August 2020

Sociodemographic characteristic	Laboratory result for COVID-19				Odds Ratio	P-value
	Negative		Positive			
	n	%	n	%		
**Age**				
Less than a year	64	12.6	11	16.9	1	0.302
1 to 5 years	80	15.8	6	9.2	0.43 [0.15 1.24]	
6 to 10 years old	150	29.6	16	24.6	0.62 [0.27 1.41]	
11 to 15 years old	213	42.0	32	49.2	0.87 [0.41 1.83]	
**Sex**						
Male	272	54.0	44	67.7	1	0.036
Feminine	232	46.0	21	32.3	0.55 [0.32 0.96]	
**Residence of the child at the time of diagnosis**						
Niamey	319	62.9	40	61.5	1	0.003
Agadez	145	28.6	14	21.5	0.77 [0.40 1.46]	
Zinder	21	4.1	2	3.1	0.75 [0.17 3.36]	
Tillabery	2	0.4	2	3.1	0.52 [0.06 3.99]	
Tahoua	10	2.0	6	9.2	4.78 [1.65 13.87]	
Dosso	8	1.6	1	1.5	0.99 [0.12, 8.17]	
Tuesday	1	0.2	0	0.0	-	
Diffa	1	0.2	0	0.0	-	

**Exposure and travel information of COVID-19 suspect children by PCR test:** history or comorbidities (OR=6.09 [1.33 27.8], p=0.03), close contact with a person with an acute respiratory infection (OR=5.08 [2.64 9.80], p<0.0001) and contact with a probable or confirmed case (OR=3.84 [2.20 6.67], p<0.0001) were statistically significant for COVID-19, unlike foreign travel, country visited and healthcare facility visited (Table 2).

**Table 2:** exposure and travel information of suspected cases according to the COVID-19 PCR test in children aged 0-15 years - Niger, February - August 2020

Patient exposure	Laboratory result for COVID-19				Odds Ratio	P value
	Negative		Positive			
	n	%	n	%		
**History or Comorbidities**						
No	503	99.2	62	95.4	1	0.03
Yes	4	0.8	3	4.6	6.09 [1.33 27.8]	
**Symptoms with the concept of travel 14 days before**						
No	461	90.9	57	87.7	1	0.08
Yes	46	9.1	8	12.3	2.01 [0.91 4.48]	
**The country visited 14 days before the symptoms**						
West Africa	20	43.5	6	75.0	1.5 [0.25 8.81]	0.65
North Africa	10	21.7	2	25.0	1	
Europe	10	21.7	0	0.0	-	
Asia	3	6.5	0	0.0	-	
America	3	6.5	0	0.0	-	
**The health center visited 14 days before the symptoms**						
No	505	99.6	64	98.5	1	0.23
Yes	2	0.4	1	1.5	3.9 [0.35 44.12]	
**Contact with a person with ARI**						
No	474	93.5	48	73.8	1	<0.0001
Yes	33	6.5	17	26.2	5.08 [2.64, 9.80]	
**Contact with a probable or confirmed case**						
No	432	85.2	39	60.0	1	<0.0001
Yes	75	14.8	26	40.0	3.84 [2.20 6.67]	

**Signs and symptoms of children suspected of having COVID-19 according to the PCR test:** the presence or absence of symptoms was significantly associated with the occurrence of COVID-19 in children aged 0 to 15 (OR=0.36 [0.21 0.61], p<0.0001) (Table 3).

**Table 3:** clinical signs and symptoms of suspected cases according to the COVID-19 PCR test in children aged 0-15 years - Niger, February - August 2020

Symptoms in cases	Laboratory results for COVID-19				Odds Ratio	P value
	Negative		Positive			
	n	%	n	%		
**Asymptomatic**						
No	109	21.5	28	43.1	1	**<0.0001**
Yes	398	78.5	37	56.9	0.36 [0.21 0.61]	
**Signs and symptoms**						
Cough	86	27.8	19	23.2	1	**0.65**
Fever	60	19.4	20	24.4	0.07 [0.03 0.14]	
Headache	22	7.1	6	7.3	1.23 [0.44 3.45]	
Sore throat	35	11.3	9	11.0	1.16 [0.48 2.82]	
Anosmia	6	1.9	3	3.7	2.26 [0.51 9.86]	
Flu	27	8.7	6	7.3	1 [0.36 2.77]	
Asthenia	18	5.8	1	1.2	0.25 [0.03 2]	
Dyspnea	28	9.1	10	12.2	1.61 [0.67 3.88]	
Conjunctivitis	2	0.6	0	0.0	-	
Others	25	8.1	8	9.8	1.44 [0.56 3.70]	

### Multivariate analysis

On multivariate analysis, the variables significantly associated with COVID-19 were: gender (ORa=0.51 [0.28 0.93], p=0.028), presence of signs or symptoms (ORa=2.29 [1.23 4.25], p=0.008) and contact with a probable or confirmed COVID-19 case (ORa=0.32 [0.13 0.77], p=0.011). We examined the performance of this logistic regression model using the Hosmer and Lemeshow test. Given that the test gives us a p-value=0.869, i.e. p-value > 0.05, then our logistic regression model is good (i.e. performs well) for predicting COVID-19 in children aged 0 to 15 who are COVID-19 suspects (Table 4).

**Table 4:** factors associated with COVID-19 in multivariate analysis in children aged 0-15 years - Niger, February - August 2020

Variables	ODD Ratio	Adjusted OR (95% CI)	P value
**Sex**			
Male	1	1	0.028
Feminine	0.55	0.51[0.28 0.93]	
**Asymptomatic**			
No	1	1	0.008
Yes	0.36	2.29[1,234.25]	
**History or Comorbidities**			
No	1		0.116
Yes	6.05	0.23[0.03 1.43]	
**Symptoms with the concept of travel 14 days before**			
No	1		0.196
Yes	2.01	0.55[0.221.36]	
**Contact with a person with Acute Respiratory Infection 14 days before symptoms**			
No	1		0.511
Yes	5.08	0.71[0.25 1.97]	
**Contact with a probable or confirmed case**			
No	1		
Yes	3.84	0.32[0.130.77]	0.011
**CI: Confidence Interval**			

## Discussion

### Children's socio-demographic characteristics

In our study, there was also a statistically significant association between the child's sex and COVID-19 (ORa =0.51; IC95% [0.28-0.93] p = 0.028). Male predominance has been reported in the literature [14-16]. The lifestyle of boys in our region exposes them more to the disease, as they are more mobile than girls, who tend to be kept at home. The prevalence of COVID-19 among suspected children was 11.36%. This result is close to that of Lu *et al*. in China, where the prevalence was 12.3% [17]. The children's ages ranged from 0 to 15 years and the median age was 10 years with [IQR: 5- 13 years] in our study, Bai *et al*. had found a median age of 11 years [IQR: 6.3- 14.5 years] [18]. Lu *et al*. lower figure than ours, with 7 [17]. Children of all ages seemed to be sensitive to COVID-19, and according to our results, the age group most affected was 11-15 years, with 42.8%. In one study, the 6-10 age group (33.9%) was the most numerous [17]. Children living in the capital Niamey were the most affected (62.8%), followed by the Agadez region (27.8%). The capital had the highest number of COVID-19 cases in the country, due to its openness to foreign countries. Agadez is a region through which a large number of migrants from neighboring countries pass. Hence the need to step up surveillance in these regions. Understanding the transmission of SARS-CoV-2 in children and their potential contribution to herd immunity is therefore essential to guide preventive strategies such as quarantine measures and school closures, but also to put an end to these imposed restrictions [19].

### Exhibition and travel information

Contact with a probable or confirmed case was associated with COVID-19 in children in our study (ORa =0.32; IC95% [0.13-0.77] p = 0.011), whereas history or comorbidities, notion of travel 14 days prior to symptoms were not. In the literature, 28% of children had a history of travel [18] 68% had been in contact with confirmed infected adults [14]. Comorbidities were found in 21% of cases [15] in study from China. Current data show that children with SARS-CoV-2 generally belong to clustered familial cases [20].

### Signs and symptoms

In our study, children with symptoms were 2.29 times more likely to be affected by COVID-19 than those without (ORa =2.29; IC95% [1.23-4.25] p = 0.008). Among the children in our study, 43.1% were asymptomatic. The percentage of asymptomatic cases varied between studies: 15.4%, 45% [17,21]. Children are often exposed to viral infections, and it is possible that they have higher levels of antibodies against the virus than adults. Another possible explanation is that children may be protected against SARS-CoV-2 because CEA-2 is less expressed at a younger age. What's more, children's immune systems are still developing, and may react to pathogens differently from those of adults. Despite this, asymptomatic cases may have a lower transmission rate but remain a major source of infection [20]. Among the clinical signs found in positive cases, fever (24.4%) and cough (23.2%) were the most predominant. Our results are in line with the findings of Du *et al*. [21] Perikleous *et al*. [22] and Zhang *et al*. [16]. The fever is explained by the inflammatory and immune response triggered by the virus, and the cough by the “diffuse alveolar damage” caused by the virus to the lung parenchyma. These lesions are encountered in many viral infections, such as influenza, or in a number of acute respiratory distress syndromes.

### Care and evolution

To prevent the spread of infection after diagnosis, home isolation for 1-2 weeks has been recommended [22]. In our study, 80% of children were monitored at home. The percentage of children hospitalized in our study was 18.5%, whereas only 5.7% of children were hospitalized due to the onset of dyspnea [20]. Children are generally asymptomatic or only mildly symptomatic, which explains the high rate of infected children followed up at home. The case-fatality rate was low in children. Infants and young children had relatively more severe disease than older children [20]. In our study, one death occurred in a 9-month-old infant, with a case-fatality rate of 1.5%. In the study by Lu *et al*., 1 death was recorded in a 10-month-old infant, and the mortality rate for children hospitalized with COVID-19 was 0.18% [16].

### Limits

As this study is retrospective, limitations are related to incomplete data and the inability to correct certain outliers. Information on parents' socio-demographic characteristics and radiographic signs in children should be collected. Due to the loss of telephone contacts, these variables were not recorded to determine whether there is an association between these characteristics and COVID-19 in children. To optimize data quality, outliers that could not be verified were removed from the final database.

## Conclusion

The COVID-19 pandemic currently represents a real challenge for healthcare worldwide. It affects children of all ages, and appears to be a benign disease. Nevertheless, children remain a major source of contamination. In our study, the most common symptoms were cough and fever. The age group most affected is between 11 and 15, with a predominance of males. Factors associated with the occurrence of COVID-19 are gender, the presence of signs or symptoms and contact with a probable or confirmed case. We recommend extending vaccination to the under-15s, and finding simple ways of applying barrier measures to children. Large-scale studies should be carried out in both health and community settings to determine the prevalence of the disease and associated factors.

### What is known about this topic

- *SARS-CoV-2 infection is less severe in children than in adults, although severe cases are reported in children;*
- *Children are a source of contamination and are predominantly male;*
- *Prevalence in Africa is poorly understood due to lack of large-scale studies*.

### What this study adds

- *This study identified the sociodemographic characteristics of children suspected of having COVID-19;*
- *It also identified factors associated with COVI-19 in children in Niger;*
- *These results can be used to draft new strategies, and their implementation will reinforce existing strategies to protect vulnerable populations (the elderly and people with co-morbidities)*.
